# The temporal asymmetry of cortical dynamics as a signature of brain states

**DOI:** 10.1038/s41598-024-74649-1

**Published:** 2024-10-16

**Authors:** Alessandra Camassa, Melody Torao-Angosto, Arnau Manasanch, Morten L. Kringelbach, Gustavo Deco, Maria V. Sanchez-Vives

**Affiliations:** 1 Institute of Biomedical Research August Pi i Sunyer (IDIBAPS), Barcelona, 08036 Spain; 2 Centre for Eudaimonia and Human Flourishing, University of Oxford, Oxford, OX3 9BX UK; 3 Department of Psychiatry, University of Oxford, Oxford, OX3 7JX UK; 4 Center for Music in the Brain, Aarhus University, Aarhus, 8000 Denmark; 5 Center for Brain and Cognition, Computational Neuroscience Group, Department of Information and Communication Technologies, Universitat Pompeu Fabra, Barcelona, 08018 Spain; 6 Institució Catalana de la Recerca i Estudis Avançats (ICREA), Barcelona, 08010 Spain

**Keywords:** Cerebral cortex, Synchronization, Oscillations, Cortical dynamics, Anesthesia, Slow waves, Sleep, Computational neuroscience, Neuroscience, Slow-wave sleep

## Abstract

The brain is a complex non-equilibrium system capable of expressing many different dynamics as well as the transitions between them. We hypothesized that the level of non-equilibrium can serve as a signature of a given brain state, which was quantified using the arrow of time (the level of irreversibility). Using this thermodynamic framework, the irreversibility of emergent cortical activity was quantified from local field potential recordings in male Lister-hooded rats at different anesthesia levels and during the sleep-wake cycle. This measure was carried out on five distinct brain states: slow-wave sleep, awake, deep anesthesia–slow waves, light anesthesia–slow waves, and microarousals. Low levels of irreversibility were associated with synchronous activity found both in deep anesthesia and slow-wave sleep states, suggesting that slow waves were the state closest to the thermodynamic equilibrium (maximum symmetry), thus requiring minimum energy. Higher levels of irreversibility were found when brain dynamics became more asynchronous, for example, in wakefulness. These changes were also reflected in the hierarchy of cortical dynamics across different cortical areas. The neural dynamics associated with different brain states were characterized by different degrees of irreversibility and hierarchy, also acting as markers of brain state transitions. This could open new routes to monitoring, controlling, and even changing brain states in health and disease.

**Supplementary Information:**

The online version contains supplementary material available at 10.1038/s41598-024-74649-1.

## Introduction

The brain expresses different states and the investigation of brain states is critical for understanding consciousness, behavior, and neurological disorders. Each transition across brain states, for example from wakefulness to sleep, brings about changes in the brain at multiple scales, from neurochemical changes leading to variation of network excitability, connectivity, and complexity, up to functional and behavioral changes^1,2^. Distinct physiological brain states—such as slow-wave sleep, rapid eye movement (REM) sleep, and wakefulness—exist. Additionally, different states within the state of wakefulness, such as resting state, or task-positive network activities^3^ such as attention, and working memory, have been extensively studied. Brain states can also be changed pharmacologically (e.g., in different anesthesia levels), and pathologically (e.g., in disorders of consciousness, epilepsy). In this context, one of the central problems in neuroscience is how to better understand the brain dynamics underlying the transitions across different brain states. Over the last few decades, much effort has been devoted to developing measures capable of quantifying the level of consciousness in humans and to describing the dynamical properties of various brain states. In particular, metrics based on observational or perturbational approaches have been widely used to characterize different brain states^1,4–9^.

Here we concentrate on a new, innovative thermodynamic framework that allows us to distinguish the features of brain dynamics with both high specificity and sensitivity^10–12^. Potentially, this could be used to find a clear signature of a given brain state by quantifying the level of non-equilibrium in brain dynamics under different brain states. Briefly, the framework uses the key hierarchical insight from thermodynamics, ‘breaking the detailed balance’, which captures the importance of the asymmetry in the directionality of information flow in non-equilibrium systems. This in turn comes straight from the second law of thermodynamics, stating that a system will go from order to disorder over time, where the level of disorder is produced by non-reversible (irreversible) processes. A non-equilibrium system is irreversible in time, while a system in equilibrium is reversible in time. This flow in a system over time can be captured by the asymmetry in the flow of events, the concept of the ‘arrow of time’^10^ and can provide a firm link between the three concepts of production entropy, non-equilibrium, and irreversibility.

Consequently, the concept of the arrow of time can be used to directly describe the hierarchical organization of the orchestration of brain computation. A well-defined arrow of time indicates a clear hierarchical organization with strong breaking of the detailed balance and asymmetric directionality of information flow, providing different computational roles to different areas. Contrary to this, a less well-defined arrow of time is indicative of more symmetry and thus a flatter hierarchy of brain organization. In other words, the level of irreversibility in brain signals can be used as a direct measure of hierarchy.

This approach has been used both in control conditions and in various neurological disorders, using human fMRI data^10^, and has more recently been applied to electrocorticography data recorded from non-human primates undergoing different states of consciousness^10,13^. Here, we describe for the first time in rodents, how the hierarchical signature, evidenced by different levels of irreversbility, is directly associated with different brain states, including both natural (sleep-awake cycle) and pharmacological (anesthesia) brain states. A crucial feature of the analysis is the use of direct neuronal population recordings which also allows not only the link with characteristic slow waves in unconscious states, but an explicit non-equilibrium characterization involving the local circuit level.

In this study we use this thermodynamic framework to describe different brain states in terms of their dynamical properties. We quantified the irreversibility of the local field potential (LFP) signals recorded under different brain states, both for spontaneous (sleep and awake), and induced by different levels of anesthesia, including a total of five brain activity patterns that were identified according to their different neuronal dynamics. Using this approach, here we provide a direct link between the neuronal dynamics associated with different brain states (e.g., synchronous and asynchronous dynamics) and the irreversibility level of the temporal evolving brain activity. We found significantly different degrees of irreversibility associated with each of the five studied states. Crucially, these differences provide evidence for differences in the hierarchy of communication of the underlying brain dynamics, opening novel routes for monitoring, controlling, and changing brain states in health and disease.

## Results

**Fig. 1:**
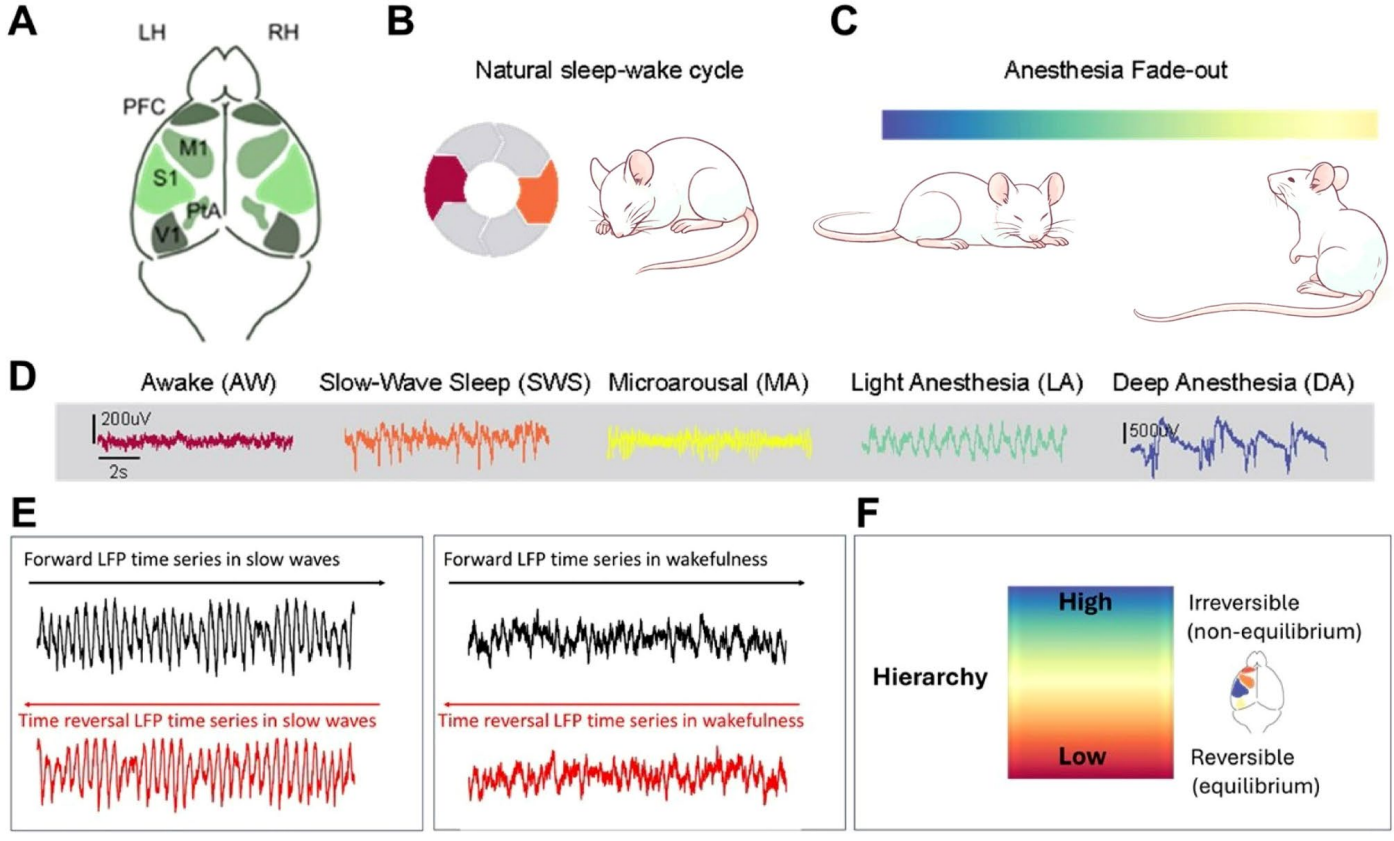
Experimental setup. (**A**) Schematic representation of the rat brain and the cortical regions in which we implanted recording electrodes (PFC, M1, S1, PtA and V1). Note that not all areas were recorded in all the subjects. (**B**) Chronically implanted rats were recorded during natural sleep-wake cycle (see Methods). (**C**) Next, the same animals underwent a protocol of anesthesia modulation where we recorded cortical activity under deep anesthesia and all the way down to wakefulness. (**D**) Example traces of the raw signal recorded from one electrode under the five different brain states that were identified and included here: awake, slow-wave sleep, microarousal, light anesthesia and deep anesthesia. (**E**) Scheme illustrating the measures of reversibility and irreversibility in slow waves (left) and wakefulness (right). (**F**) Hierarchy based on the degree of reversibility (irreversibility).

**Fig. 2:**
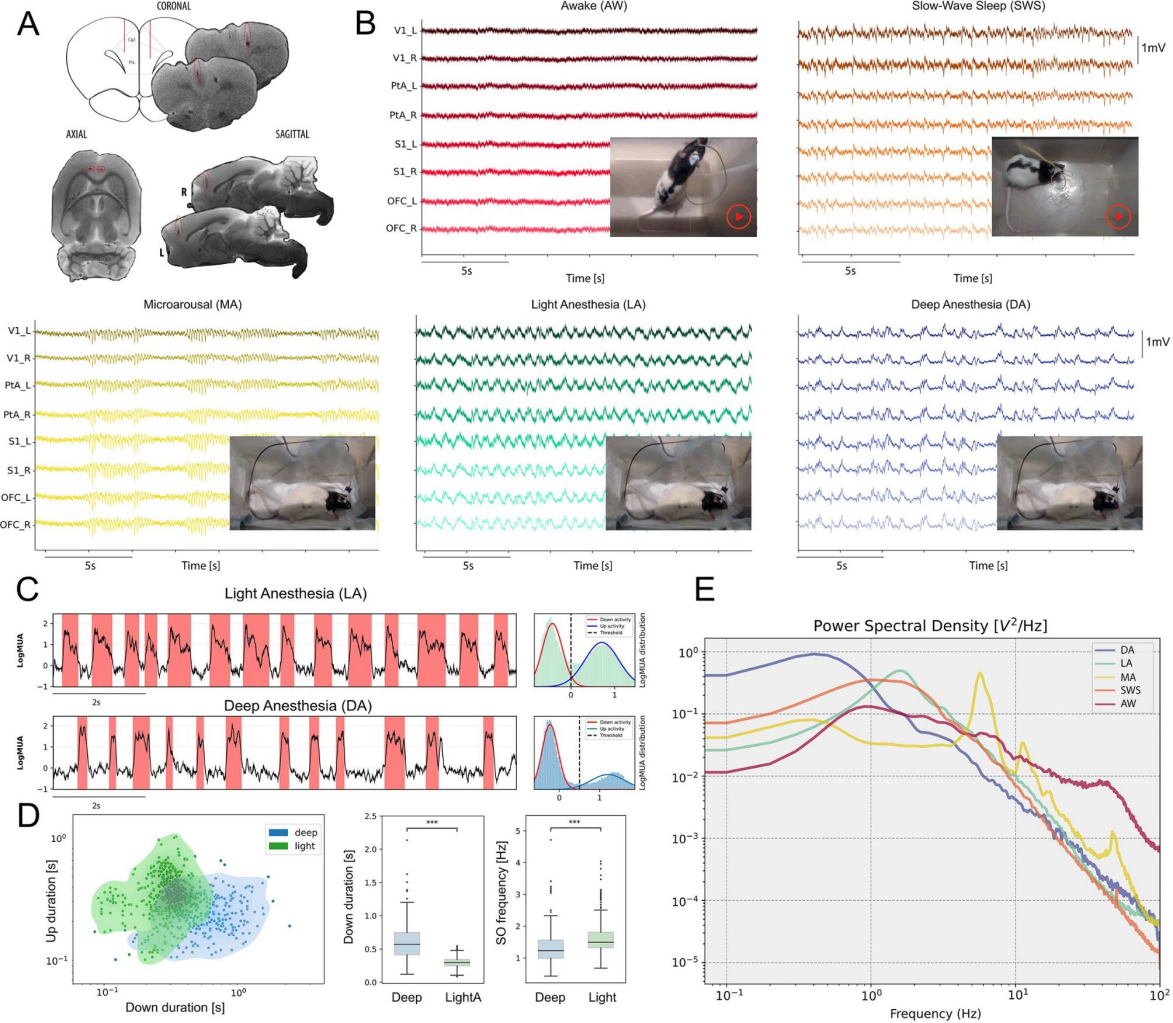
Electrode localization, cortical activity under different brain states and brain states identification criteria. (**A**) Example showing the equivalence of the coronal slices with the atlas^53^. Marked in red is the deepest electrode trajectory. (**B**) Activity of eight cortical channels implanted in the left and right hemispheres under awake state (red), slow-wave sleep (orange), and in three transient states occurring during anesthesia fade out: microarousal (yellow), light anesthesia (green) and deep anesthesia (blue). (**C**) Detection of Deep (DA) and Light (LA) slow-wave anesthesia states according to the features of slow wave activity. The detection of the Up and Down states was performed on the multiunit activity that showed a bimodal distribution (C, right hand side panel). (**D**) The distribution of the Up and Down state durations, where the deep anesthesia state is characterized by significantly longer down states and a lower frequency of slow oscillations (Wilcoxon rank sum, ***p < 0.0001). AW (awake), SWS (slow-wave sleep), MA (microarousals), LA (light anesthesia), DA (deep anesthesia). (**E**) PSD illustrating the main spectral features of the five brain states included in the study.

Cortical electrophysiological recordings were collected from rats with chronic implants, located as depicted in Fig. 1A. Two main strategies were employed to obtain recordings from different brain states: (1) the physiological states of quiet wakefulness (i.e., awake, AW) and slow-wave sleep (SWS) were extracted from all subjects (*N* = 5 rats) during their natural sleep-wake cycle (Fig. 1B); (2) the same subjects underwent a fade-out anesthesia protocol (Fig. 1C), where we induced a deep anesthesia state with an intraperitoneal injection of a mixture of ketamine and medetomidine, and recorded the electrophysiological activity until the animals woke up (Fig. 2) as described by Tort-Colet et al.^14^ Previous studies have shown that the different brain states that can be obtained through anesthesia modulation are characterized by different dynamical properties and different levels of cortical complexity (e.g., Brown et al.^15^). We have previously used this approach and found that it is possible to identify different states with characteristic dynamical features as they spontaneously emerge from anesthesia^1,14,16^. Further, for sleep stages, a combination of cortical activity pattern, electromyography (EMG) and behavior was used (see Materials and Methods). Accordingly, we included in our analysis the two physiological states mentioned above (SWS and wakefulness) and three more transient brain states induced by the anesthesia, labelled deep anesthesia–slow waves (DA), light anesthesia–slow waves (LA) and microarousals (MA) following the previous definition by Tort-Colet et al.^14^. The period of microarousals is transitory between deep anesthesia and wakefulness, and contains periods of synchronization (slow waves) and periods of desynchronization.

Periods of wakefulness and SWS periods were classified based on local field potential (LFP), EMG, and video following the scale for sleep scoring from Silber et al.^17^. The signals from awake periods were asynchronous and had low amplitudes with an active muscle tone. SWS exhibited a higher amplitude signal and a slow oscillatory pattern of Up/Down states (0.2–3.5 Hz) with a diminished muscle tone.

Deep anesthesia exhibited a very slow oscillatory pattern with maximal amplitude and long-lasting Down states (0.6 ± 0.28 s). Light anesthesia was marked by shorter Up (0.37 ± 0.15 s) and Down states (0.29 ± 0.08 s) and higher frequency of slow oscillations (1.63 ± 0.52 Hz). Microarousals emerged when the anesthetic effect was fading out and were characterized by a desynchronized activity pattern lasting 5–20 s, interspersed with a fast oscillation (6–7 Hz) lasting 4.5 ± 2 s, as described by Tort-Colet et al.^14^. All the states used here were characterized by different dynamical features and spectral components (see Methods).

Departing from the idea that living matter is characterized by non-equilibrium dynamics, here we used a theoretical framework able to extract the level of irreversibility of brain signals. Such a framework provides the possibility of estimating how the external, extrinsic environment drives internal, intrinsic brain dynamics. Briefly, the measure previously developed by Deco et al.^10^ relies on the calculation of the arrow of time of cortical activity through the estimation of the degree of asymmetry obtained by comparing the causal relationship between pairwise time series: the forward activity recorded from the brain, and the artificially generated reversed backward version (Fig. 1E,F; for details, see Materials and Methods and Perl et al.^13^). The arrow of time, measured as the asymmetry in the signal, will define a hierarchy across areas for each brain state, the lowest being highly symmetric and thus, reversible. If an area is expressing a highly reversible signal, it suggests a low computational value for information processing. The opposite is the case for high irreversibility.

Once the different brain states were classified, for the reversibility/irreversibility analysis of the LFP, for each subject and each brain state we used five segments of activity with a variable duration ranging between 10 and 200 s (Fig. 2). All the available data segments for each brain state were further divided into 10-s windows and analyzed with the INSIDEOUT framework described by Deco et al.^10^. In this way, we obtained 301 windows of AW, 178 windows of SWS, 728 windows of MA, 685 windows of LA, and 527 windows of DA.

**Fig. 3:**
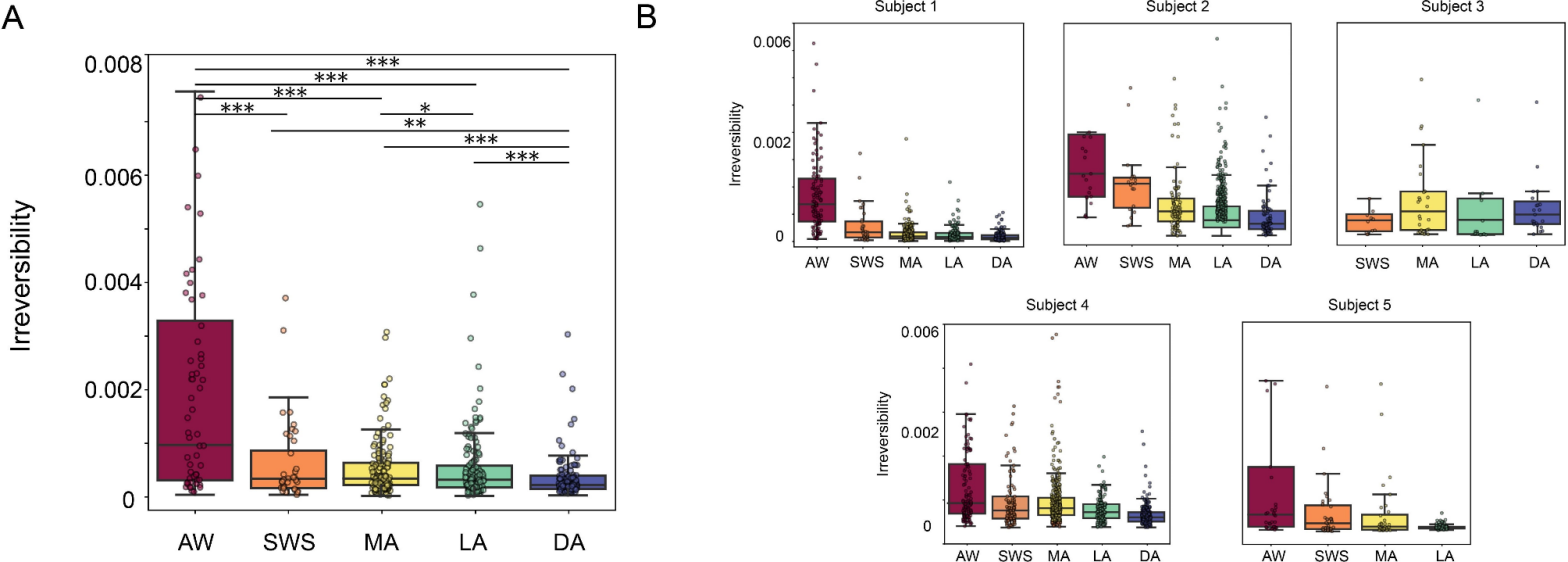
Irreversibility of cortical dynamics under different brain states. (**A**) Boxplot of the values of irreversibility obtained from all the subjects. Each dot represents the irreversibility value of a 10-s window extracted from the corresponding brain state. (Wilcoxon rank sum, *p < 0.01, **p < 0.001, ***p < 0.0001). (**B**) Boxplot of the values of irreversibility computed on each of the five subjects. Notice that subject 3 was not recorded in awake state, while subject 5 was not recorded under deep anesthesia. All subjects apart from subject 3 show the same trend as the population results. AW (awake), SWS (slow-wave sleep), MA (microarousals), LA (light anesthesia), DA (deep anesthesia).

In Fig. 3A, we show the distribution of irreversibility values under each brain state at the population level using boxplots. Also, the results for each single subject are shown in Fig. 3B. Group-level comparisons were made, showing a statistically significant difference of irreversibility (Table 1) and hierarchy (Table 2) across most brain states (Wilcoxon rank sum, *p < 0.01, **p < 0.001, ***p < 0.0001, Table 1).

**Table 1:** Table of *p*-values obtained computing the statistical differences between irreversibility values under different brain states obtained using the Wilcoxon rank sum test.

Irreversibility	AW	SWS	MA	LA	DA
AW		*p* = 1.29e−15	*p* = 1.01e−26	*p* = 4.001e−32	*p* = 7.55e−44
SWS	*p* = 1.29e−15		*p* = 0.2	*p* = 0.71	*p* = 0.0004
MA	*p* = 1.01e−26	*p* = 0.21		*p* = 0.0066	*p* = 1.47e−18
LA	*p* = 4.001e−32	*p* = 0.71	*p* = 0.0066		*p* = 1.31e−08
DA	*p* = 7.55e−44	*p* = 0.0004	*p* = 1.47e−18	*p* = 1.31e−08	

**Table 2:** Table of *p*-values obtained computing the statistical differences between hierarchy values under different brain states obtained using the Wilcoxon rank sum test.

Hierarchy	AW	SWS	MA	LA	DA
AW		*p* = 1.11e−12	*p* = 1.64e−22	*p* = 6.3e−30	*p* = 5.18e−39
SWS	*p* = 1.11e−12		*p* = 0.28	*p* = 0.19	*p* = 0.00015
MA	*p* = 1.64e−22	*p* = 0.28		*p* = 0.0005	*p* = 8.05e−16
LA	*p* = 6.3e−30	*p* = 0.19	*p* = 0.0005		*p* = 1.9e−05
DA	*p* = 5.18e−39	*p* = 0.00015	*p* = 8.05e−16	*p* = 1.9e−05	

**Fig. 4:**
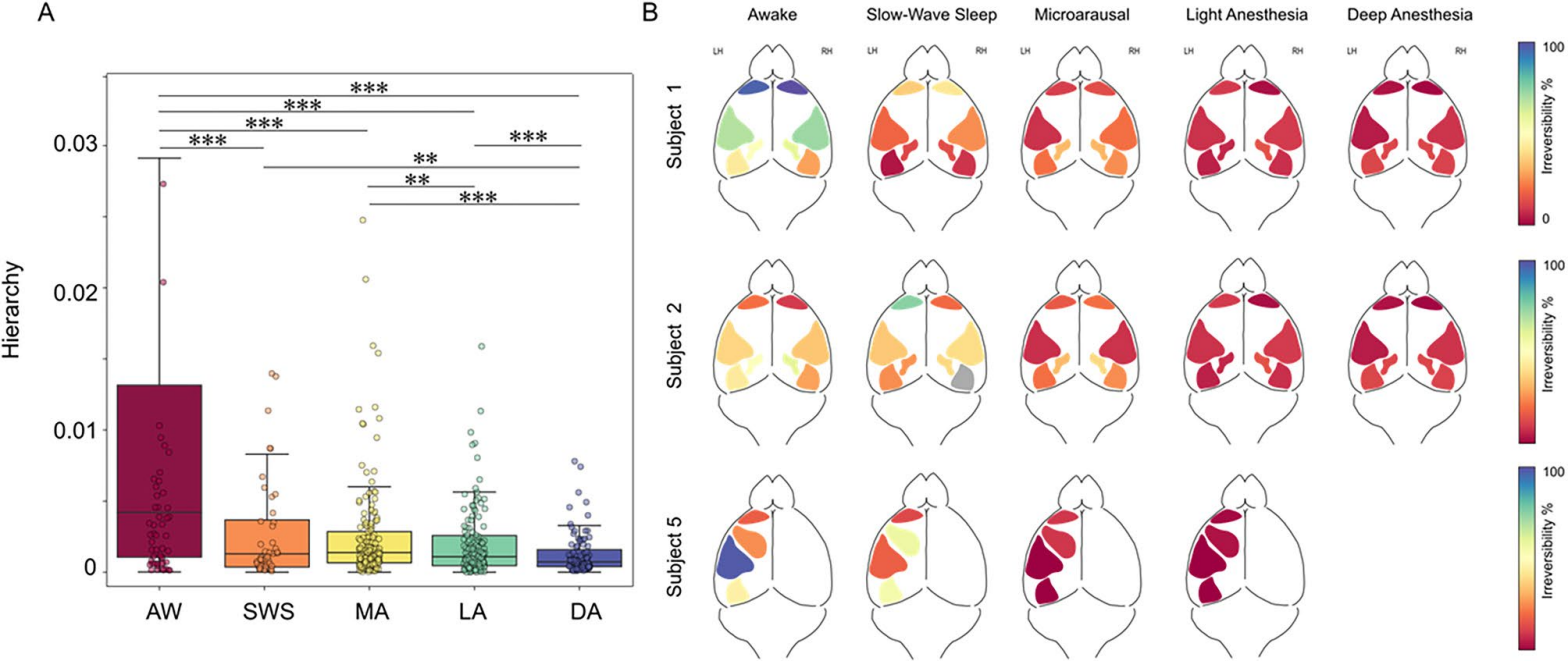
Hierarchy of cortical dynamics under different brain states. (**A**) Boxplot of the values of hierarchy obtained from all the subjects as the standard deviation of the irreversibility values across areas. Each dot represents the hierarchy value computed on a 10-s window extracted from the corresponding brain state. (Wilcoxon rank sum, *p < 0.01, **p < 0.001, ***p < 0.0001). (**B**) Macroscopic maps showing the irreversibility per cortical region under each brain state. Notice that while subjects 1 and 2 have bihemispheric recording, in subject 5 the recording electrodes were implanted only in the Left hemisphere. AW (awake), SWS (slow-wave sleep), MA (microarousals), LA (light anesthesia), DA (deep anesthesia).

To estimate the level of orchestration in the brain and its relationship to the state of awareness, we computed here a measure of hierarchy. Hierarchy was defined as the variability of the level of irreversibility across different cortical areas, according to the definition provided by Deco et al.^10^ (see Materials and Methods). At the population level, the hierarchy values also showed a dependence on the level of consciousness, being high in the awake state and progressively decreasing when moving towards more unconscious brain states (Fig. 4). Moreover, a dynamical difference (Table 1) between slow-wave activity under anesthesia (LA, DA) and SWS was highlighted by the macroscopic maps of irreversibility (Fig. 4B). In Fig. 4B we show the macroscopic maps of irreversibility for three subjects. While subject 1 and 2 had the same cortical regions recorded, subject 5 had a different recording setup (see Materials and Methods for details). Despite the difference in the areas recorded, the dynamical behavior denoted under the different brain states was consistent across subjects. Indeed, in all of them SWS was characterized by higher hierarchy values than DA states (Fig. 4A) and by higher variability of irreversibility values across areas (Fig. 4B).

**Fig. 5:**
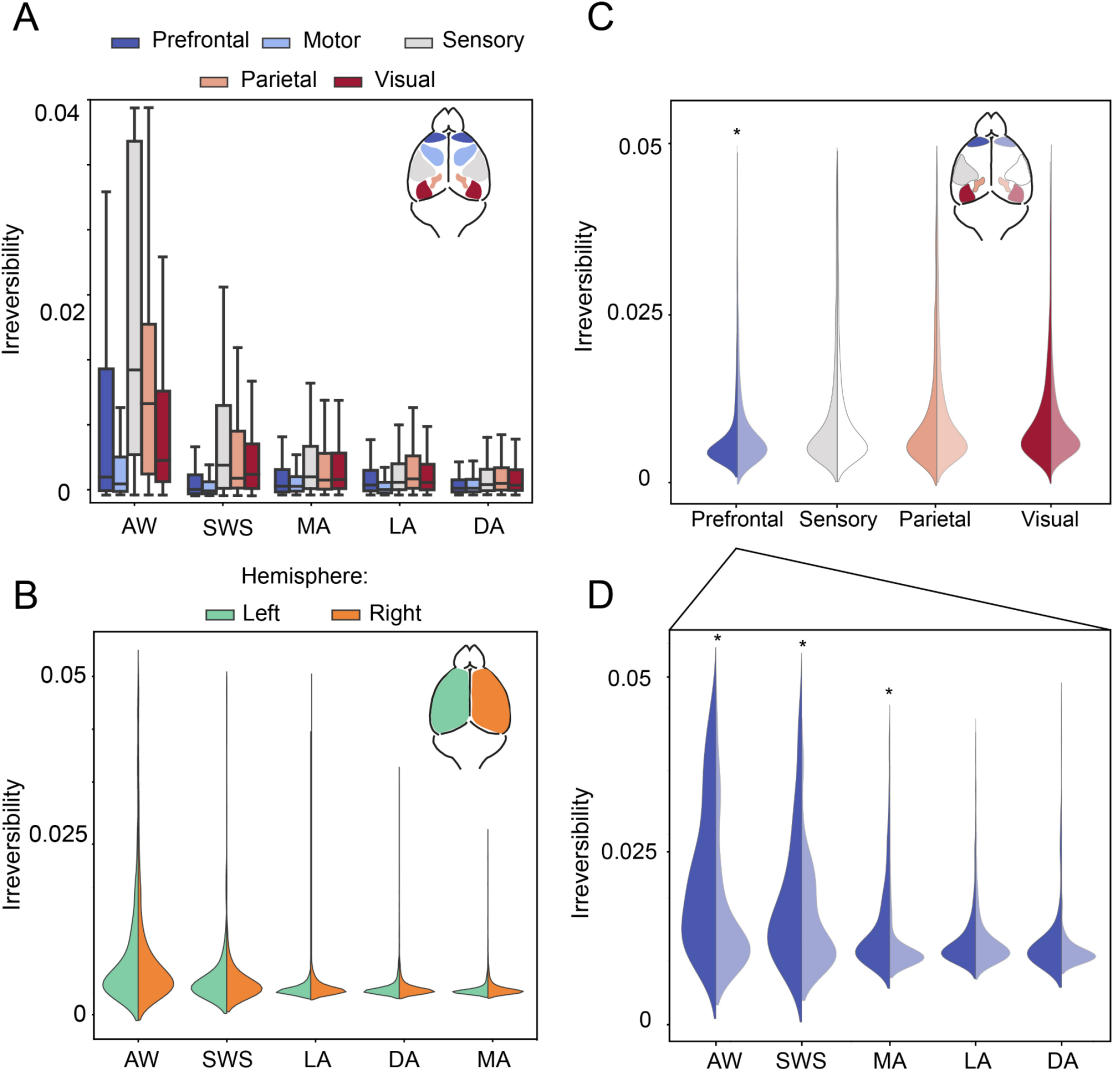
Irreversibility across different cortical areas. (**A**) Boxplot of the values of irreversibility for each cortical region under different brain states at the population level (*N* = 5). (**B**) Irreversibility of the left and right hemisphere under different brain states (*N* = 2). (**C**) Irreversibility of each cortical region in the left and right hemisphere (*N* = 2) where the prefrontal region showed a significant interhemispheric difference. *p < 0.05. (**D**) Irreversibility of prefrontal region under different brain states showed significant interhemispheric difference in AW, SWS and MA states. AW (awake), SWS (slow-wave sleep), MA (microarousals), LA (light anesthesia), DA (deep anesthesia). (Wilcoxon rank sum, *p < 0.05)

The anatomical reconstruction performed in all the subjects allowed us to ascertain the exact location of each recording electrode, and so the precise cortical region from which we were recording. As such, we were able to perform a group-level comparison of the irreversibility level under different brain states across areas. Prefrontal and motor areas showed on average a lower irreversibility value with respect to the other areas (Fig. 5A) in all brain states (Tables 3, 4, 5, 6 and 7). Also, we observed a high variability of irreversibility in the prefrontal cortex (PFC) across subjects during wakefulness (Fig. 4B). No significant differences between the irreversibility in the left and right hemispheres were observed across all brain states at the population level (Fig. 5B, *N* = 2 rats implanted in both hemispheres). Nevertheless, looking at single areas, we found a significant difference across hemispheres in the PFC (Fig. 5C) in the AW, SWS, and MA states (Fig. 5D). These differences were damped under deeply unconscious anesthesia states where all areas showed a homogeneous level of irreversibility both across areas and across hemispheres, leading to no significant differences for LA and DA (Fig. 5D). Overall, our results show that a larger breaking of the balance (i.e., a higher hierarchy value), is associated with a larger level of irreversibility and therefore with a higher level of consciousness.

**Table 3:** Table of *p*-values of the statistical differences between cortical areas in awake state obtained using the Wilcoxon rank sum test.

AWAKE	Motor	Parietal	Prefrontal	Sensory	Visual
Motor		1.35e−33	2.27e−04	1.38e−53	2.76e−20
Parietal	1.35e−33		1.12e−17	1.41e−11	1.31e−08
Prefrontal	2.27e−04	1.12e−17		2.34e−40	3.91e−07
Sensory	1.38e−53	1.41e−11	2.34e−40		5.21e−33
Visual	2.76e−20	1.31e−08	3.91e−07	5.21e−33	

**Table 4:** Table of *p*-values of the statistical differences between cortical areas in slow wave sleep state obtained using the Wilcoxon rank sum test.

SWS	Motor	Parietal	Prefrontal	Sensory	Visual
Motor		1.24e−18	0.19	4.99e−25	2.98e−22
Parietal	1.24e−18		2.8e−13	0.027	0.94
Prefrontal	0.19	2.8e−13		3.26e−19	9.7e−16
Sensory	4.99e−25	0.027	3.26e−19		0.013
Visual	2.98e−22	0.94	9.7e−16	0.013	

**Table 5:** Table of *p*-values of the statistical differences between cortical areas in microarousal state obtained using the Wilcoxon rank sum test.

MA	Motor	Parietal	Prefrontal	Sensory	Visual
Motor		1.65e−21	0.18	6.43e−31	3.63e−27
Parietal	1.65e−21		2.65e−20	8.36e−03	0.37
Prefrontal	0.18	2.65e−20		1.17e−30	2.45e−26
Sensory	6.43e−31	8.36e−03	1.17e−30		0.048
Visual	3.63e−27	0.37	2.45e−26	0.048	

**Table 6:** Table of *p*-values of the statistical differences between cortical areas in light anesthesia state obtained using the Wilcoxon rank sum test.

LA	Motor	Parietal	Prefrontal	Sensory	Visual
Motor		5.16e−43	6.98e−16	2.76e−31	1.58e−26
Parietal	5.16e−43		7.5eE−24	2.21e−06	2.3e−07
Prefrontal	6.98e−16	7.53e−24		1.55e−09	3.66e−06
Sensory	2.76e−31	2.21e−06	1.55e−09		0.36
Visual	1.58e−26	2.3e−07	3.66e−06	0.36	

**Table 7:** Table of *p*-values of the statistical differences between cortical areas in deep anesthesia state obtained using the Wilcoxon rank sum test.

DA	Motor	Parietal	Prefrontal	Sensory	Visual
Motor		7.19e−15	0.646	2.33e−10	4.13e−09
Parietal	7.19e−15		6.37e−24	1.07e−01	0.028
Prefrontal	0.646	6.37e−24		1.38e−16	6.97e−15
Sensory	2.33e−10	1.07e−01	1.38e−16		0.58
Visual	4.13e−09	2.8e−020.028	6.97e−15	0.58	

## Discussion

We investigated the irreversibility of emergent cortical dynamics in five different brain states that correspond to different levels of information processing, ranging from wakefulness to SWS and deep anesthesia. Wakefulness, a state characterized by asynchronous patterns^18–21^ and complex interactions, resulted in the highest irreversibility of dynamics, which can be considered a signature of this state. Further, we found a spatial correlate, namely the state with highest heterogeneity in reversibility across areas. Irreversibility decreased towards more synchronous patterns, with a minimum level in deep anesthesia, while homogeneity in reversibility becomes the spatial signature of these states. In conclusion, different brain states are characterized by the asymmetry of the underlying causal interactions between pairs of regions, given that the level of irreversibility captures the breaking of the underlying detailed balance.

Other studies have used different measures of entropy to classify brain states, a measure from information theory that quantifies the randomness in a system. In brain dynamics, entropy measures of spontaneous activity quantify the amount of information produced over time, and can indicate the complexity variability of brain states^22–24^. Although both irreversibility and entropy can be used to identify brain states, irreversibility focuses on directional asymmetry in brain signals, while entropy measures the randomness and information production in brain signals.

The objective here is not to improve or replace other classification methods, but to offer a deeper understanding of the cortical network dynamics in different states and the origin of wakefulness. Overall, the results establish a direct link between nonequilibrium dynamics, irreversibility of time signals, hierarchy of interactions, and the emergence of wakefulness.

### A theoretical framework for estimating cortical irreversibility

Over the last few decades, the neuroscience community has made significant efforts to understand and extract the dynamical signatures of different brain states. The brain can be seen as a non-linear system outside of equilibrium, driven by complex intrinsic and extrinsic forces. Adopting the model of coupled phase oscillators proposed by Kuramoto in 1984^25^, the intrinsic forces ruling brain dynamics can be explained in a simplified form. They represent the forces of interaction between coupled pairs of neural oscillators following the rules imposed by brain connectivity and by the neural coupling interaction functions^26–28^. The extrinsic forces driving the brain are related to the brain’s interaction with the environment^29–31^. According to these ideas, a living system such as the brain requires the breaking of the balance between intrinsic and extrinsic forces in order to operate in an optimal highly complex regime, such as the dynamic asynchronous regime characterizing natural conscious brain states. Following these ideas, and using a computational framework recently developed by Deco et al.^10^ here we provide a characterization of several brain states observed in rats during their natural sleep-wake cycle and across different levels of anesthesia^1,16,32,33^. The impact of extrinsic forces has also been discussed in the field of non-autonomous dynamical systems^34–36^, and also in relation to biological and neural systems^37^, albeit here we instead focus on the use of thermodynamics for inferring the functional hierarchical orchestration of the underlying dynamics in different states. Thermodynamics provides a general framework for studying hierarchy in both physical and biological systems. Indeed, nonequilibrium quantifies the asymmetry of information flow by estimating the reversibility and non-reversibility (irreversibility) over time of the underlying processes.

### Cortical irreversibility and neural dynamics

Here we used a theoretical framework^10^ that revealed significantly different levels of irreversibility and hierarchy associated with different brain states (awake, SWS, and anesthesia) using LFP recordings from non-human primates, differences that were previously rarely captured using conventional methods (e.g., functional connectivity methods). Here we applied this measure to LFP recordings from chronically implanted rats during their natural sleep-wake cycle and during different levels of anesthesia all the way to wakefulness. In contrast to previous work, which has assessed quantitatively brain states using metrics based on the analysis of the spatiotemporal neural dynamics such as complexity, wave propagation, entropy, or integrated information^1,21,23,38^, here we used irreversibility and hierarchy measures to characterize different brain states. Our results show that wakefulness (AW) emerges as the state with the highest irreversibility and highest diversity of values across areas, while deep anesthesia was characterized by the lowest level of irreversibility. These results can be framed in the context of studies describing the slow cortical rhythms occurring under unconscious brain states as dynamical states dominated by synchronous neural activity^39–41^. This synchronous activity, characterized by a rhythm known as slow wave activity (SWA) or slow oscillations (SOs), has been proposed to be the default activity mode of cortical circuits^42,43^. Such activity patterns emerge as a result of two main components: cortical recurrency and adaptation^44–46^. Indeed, in a low-dimensional representation of brain network behavior provided by mean-field theory^45^, the dynamical regime of SO occupies a wide region of the excitation/adaptation diagram, meaning that such states can be expressed by multiple network configurations and multiple combinations of parameters^43^. A central SO region corresponds to the most regular oscillations and also with a deep valley in the energy landscape (see Fig. 2C in Sanchez-Vives et al.^43^), which is to say that a minimum amount of energy is needed for the brain system to stay in such a dynamical state characterized by synchronous neural dynamics. Hence, we could argue that this minimum energy can only be maintained in a dynamical state that lies close to the thermodynamic equilibrium associated with maximum reversibility; that is, the state that the cortical network tends to express or its default activity pattern^42,43^.

### Cortical irreversibility in transient states

Here we wanted to shed light on the less-studied transient states that the brain system visits during anesthesia fade out (microarousals, light anesthesia, and deep anesthesia) which have been previously characterized^14,16^ and that we have identified in our data through spatiotemporal analysis of the neural dynamics in LFP recordings. We compared the level of irreversibility associated with the transient brain states with that associated with the global brain states of wakefulness and SWS detected during the rats’ natural sleep-wake cycle. Our hypothesis was that transient states may be characterized by different levels of irreversibility and that the irreversibility of each of those states will increase proportionally to the level of awareness while anesthesia fades out and the system approaches the awake state. Transient states characterized by different frequencies of SO under anesthesia show changes in collective dynamics that are accompanied by, but not limited to, modulations of cortical excitability, wave propagation^38^, functional connectivity, and cortical complexity^1,21^. Also, it is known that moving from SO regimes towards wakefulness the cerebral cortex entrains in another oscillatory rhythm: the microarousal state^47,48^. Tort-Colet et al. described that the microarousal state emerging during fading out of anesthesia is characterized by a rhythmic alternation between synchronous and asynchronous periods, with different activation levels and spectral content with respect to the SO states, and so by different dynamical underpinnings^14^. We considered three global brain states: wakefulness, sleep, and anesthesia. Accordingly, we detected here in the recordings performed under anesthesia three different transient states labelled as deep anesthesia (DA), light anesthesia (LA), and microarousal (MA), and computed the irreversibility value for each of them. Our results confirm the previously reported observations and our hypothesis: the three states are characterized by different degrees of irreversibility, which increases progressively from DA to MA, proportionally to the level of arousal. Even when the microarousal states contain desynchronized periods (which would suggest that the irreversibility would be closer to wakefulness), the highly synchronized interspersed periods^14^ in this state seem to prevent such take-off. Microarousal periods therefore deserve a deeper future analysis from a cortical dynamics perspective.

### Cortical irreversibility across cortical areas

As a result of the anatomical reconstruction performed in this study, we were able to compute the level of irreversibility of cortical activity recorded from different cortical areas under different states of awareness. We quantified the area-specific dynamical changes due to brain state transitions, as well as the variability of irreversibility across areas related to natural and induced brain states (i.e., a hierarchy measure). Interestingly, besides the modulation of the average irreversibility observed under different brain states, differences emerged between cortical areas in all conditions. In particular, we found that a lower level of irreversibility is associated with prefrontal and motor areas, while higher irreversibility was found in sensory areas that are more extrinsically driven by the environment in AW, SWS and MA states. Such differences in the degree of irreversibility associated with different areas may be due to the heterogeneity of the innervation along the rostro-caudal axis^49^ which in turn generates differences in the excitability level of different cortical areas, leading to variable dynamical patterns of activity that are detectable measuring cortical irreversibility. Interestingly, the PFC displayed significant differences in irreversibility across hemispheres during wakefulness. Even when we cannot rule out micro-scale differences in electrode location, prefrontal lateralization has been described in rats (albeit lesser than in humans) that could be a contributing factor. We found different levels of hierarchy associated with both global brain states (i.e. wakefulness, sleep and anesthesia) and transient states (i.e. MA, LA, DA), the awake state being the one characterized by higher variability and deep anesthesia the state with lower hierarchy values. Moreover, in the transient states we observed a modulation of the hierarchy which suggests that a progressive spatiotemporal modulation of the dynamical features of the cerebral cortex occurs during anesthesia fade-out to recover the network properties needed to build up conscious behavior.

We investigated in detail SWS and different levels of anesthesia showing a bistable dynamic (DA and LA), that we previously characterized in the rat^14,50^ and mouse^1^. Interestingly, the temporal features of slow waves in SWS have been described to be closer to those in light anesthesia than in deep anesthesia^50^. We were able here to compare the hierarchy of the dynamics of SWA recorded under SWS and light and deep anesthesia in the same subjects. The macroscopic maps showing the irreversibility values across different areas showed a quite homogeneous causal driving of the cortical regions in all anesthesia conditions (MA, LA, DA). This is not the case for sleep, where the different regions showed a variable distribution of irreversibility values, similar to that observed in the awake condition. So, when looking at the macroscopic maps of irreversibility under SWS (Fig. 4B), we found a large heterogeneity which in this case contrasts with both deep and light anesthesia. We speculate that a certain degree of richness of cortical dynamics is preserved during SWS to allow the abrupt and fast recovery of complex conscious behavior that is needed to wake up.

Overall, our results, together with those previously published with different measures^10,13,51,52^, open the possibility of extrapolating features related to the modulation of interactions between cortical areas occurring during the transition between conscious and unconscious brain states, through the estimation of the arrow of time of brain signals which strongly correlates with the state of awareness. This establishes a direct link between synchronous and asynchronous neural dynamics associated with different brain states and their underlying dynamical properties.

## Materials and methods

### Experimental design

#### Animals and chronic implants

For these experiments, five male Lister-hooded rats (250–450 g, 6–10 months old) were chronically recorded during both their natural sleep-wake cycle and during anesthesia using implanted electrodes (see below). All experiments were carried out in accordance with Spanish regulatory laws (BOE-A-2013-1337), which comply with European Union guidelines on the protection of animals used for scientific purposes (Directive 2010/63/EU and of the council of 22 September 2010) and were evaluated and approved by the Animal Experimentation Ethics Committee (CEEA) of the University of Barcelona (287/17 P3). The study is reported in accordance with ARRIVE guidelines.

The implant consisted of a case blank connector with crimping contacts (Molex, Lisle, USA) to attach the bipolar electrodes (100 μm thickness of insulated stainless steel, California Fine Wire Co, Grover Beach, USA), which were twisted and with a tip separation of 400 μm The electrodes were surgically implanted bilaterally (*N* = 2) or in the left hemisphere only (*N* = 3) in five different cortical regions. Using a stereotaxic apparatus (Kopf Instruments, Tujunga, USA) the following coordinates for each region^53^ were reached: Prefrontal cortex (PFC) (2.7 AP, ± 0.5 ML, − 2.8 DV), primary motor M1 (0.20 AP, ± 1.6 ML, − 1 DV), primary somatosensory S1 (− 1.8 AP, ± 4.3 ML, − 1.3 DV), parietal association area PtA (− 3.6 AP, ± 2 ML, − 0.8 DV) and primary visual V1 (− 6.6 AP, ± 3.5 ML, − 0.9 DV). The PFC and V1 were recorded from the five animals, S1 and PtA from three and M1 from two of them. A single insulated Tungsten wire (125 μm, Advent Research Materials Ltd, Oxford, UK) was also implanted in the neck muscle for the recording of EMG during the sleep-wake cycle. Further details of the surgical procedure can be consulted in Torao-Angosto et al.^50^. For a minimum of 5 days after surgery the animals were monitored to ensure their correct recovery, being daily treated for analgesia and possible infections with buprenorphine (0.06 mg/kg) and enrofloxacin (25 mg/kg), respectively. At the conclusion of the chronic recordings, euthanasia was induced via an injection of sodium pentobarbital (200 mg/ml, Dolethal^®^), followed by a transcardiac perfusion after which the brain was carefully extracted and fixed in 4% paraformaldehyde.

#### Recording protocols

After the post-surgical recovery, the animals were handled and habituated to the experimenter and the recording cage (57 × 39 × 42 cm) for a minimum of 5 days to minimize stress during the experiments. In the experimental sessions, the local field potentials (LFPs) from the freely moving animals were recorded first during their natural sleep-wake cycle and then during different levels of anesthesia. The subjects were placed in a plastic cage inside an acoustic isolation box, and then connected to a headstage micro preamplifier (Multi Channel Systems, Reutlingen, Germany) using a custom-made adapter (IMB-CNM, CSIC). All sessions were videotaped, and the brain signals were acquired and digitized at 10 kHz using a data acquisition interface and Spike 2 software (Cambridge Electronic Design, Cambridge, UK).

#### Sleep-wake cycle

The subjects were recorded for a minimum of 3 h daily during their natural sleep-wake cycle.

#### Anesthesia

After a baseline recording with the animal in its physiological awake state, a mixture of ketamine (Ketolar 50 mg/ml) and medetomidine (Domtor 1 mg/ml) was administered by intraperitoneal injection. Doses ranging from 20 to 80 mg/kg of ketamine and from 0.15 to 1 mg/kg of medetomidine were used to elicit a deep state of anesthesia. Cortical activity was recorded starting from induction, during the slow oscillatory period of anesthesia until the complete fade-out of the anesthesia, up to wakefulness, using a strategy that we have previously developed^1,14,16^. Deep anesthesia exhibited a slow oscillatory pattern with maximal amplitude and long-lasting Down states^14^. When Down states or silent periods in between waves are long, some authors refer to it as a burst suppression state^54–56^. However, we do not use this term here because the frequency of slow waves progressively decreases for deeper anesthesia in a continuum^1^. Light anesthesia was marked by shorter Down states and higher frequency of slow oscillations (Fig. 2A, C, D). Microarousals emerged when the anesthetic effect was fading out and were characterized by a desynchronized activity pattern interspersed with a fast oscillation, as described by Tort-Colet et al.^14^. The precise criteria are described in the following section.

### Data analysis for brain state detection

The different brain states have been detected based on criteria derived from our previous studies on brain state characterization^1,14,16,50^, where both the temporal and spectral features of the neural dynamics are taken into account.

The brain states identified in the anesthesia protocol were defined as follows. The DA state (deep anesthesia-slow waves) is that occurring following induction of anesthesia (Fig. 2F). The DA state displays high-amplitude (> 500 uV) and low-frequency (< 1.25 Hz; Fig. 2) slow oscillations^1^. As anesthesia fades out, the period of LA (light anesthesia-slow waves; Fig. 2B) starts, the slow oscillation becomes more regular and the duration of the Down states decrease, thus increasing the frequency of the oscillatory cycle (Fig. 2C–E^1,14^).

The new state in the transition towards wakefulness is what we refer to as the microarousal state (MA; Fig. 2B)^14^. It consists of an alternation between synchronous periods of delta/theta waves (4–8 Hz, Fig. 2B) and large periods of asynchronized activity, with both substates being larger than 2 s. As anesthesia fades out, the synchronous periods of the MA state start to vanish and only the asynchronous activity remains. When anesthesia is completely faded out, the awake (AW; Fig. 2B) state can be detected. The AW state is characterized by an asynchronous low-amplitude (< 200 uV) and high frequency in the gamma band trace (30–100 Hz, Figs. 1D and 2B and E) which is accompanied by high activity in the EMG signal due to the animal moving (not shown), this being a sign that the animal is awake. We selected awake periods from three different conditions: pre-anesthesia recordings, post-anesthesia recordings with the animal moving, and recordings from the sleep-wake cycle. We found no significant differences in irreversibility and hierarchy among these three wakefulness periods.

The natural slow-wave-sleep state (SWS; Fig. 2C) displays similar features to the LA state, as shown by Torao-Angosto et al.^50^. It is characterized by high amplitude (> 500 uV) low-frequency (> 1.25 Hz, Fig. 2B) slow oscillations with Ups and Down states smaller than 0.5 s. These features distinguish it from REM or wakefulness states present in the sleep-wake cycle.

#### Detection of up and down states

The detection of the transitions from silent (Down) to active (Up) states, and vice-versa, was performed using a *z*-scored normalized multivariate time series, composed of the raw signal (LFP), a log-scaled 200–1500 Hz power estimation of the LFP (the multi-unit activity, MUA) (based on Mattia and Del Giudice^57^ and used in Reig et al.^58^, Ruiz-Mejias et al.^59^ and Dasilva et al.^1^) and envelope of the variance of the gamma-filtered LFP^60^.

Principal Component Analysis (PCA) was applied to the time series and a bimodal distribution was obtained from the projections over the first principal component. The peaks of the distribution represent samples of the projection, and thus the LFP that belongs to either silent or active states. This bimodal distribution (Fig. 2C, panels to the right) allowed us to select a threshold that optimally discriminated between Up and Down samples. The minimum duration for each state was set to 80 ms to avoid detecting random fluctuations of the signal.

#### Spectral analysis

The traces for each brain state were divided into periods of 100 s and their power spectral density (PSD) was computed using Welch’s method from scipy (scipy.org, signal.welch) with a 3-s window and 0.1-Hz bin size.

#### Anatomical reconstruction

The correct placement of the electrodes was determined using magnetic resonance imaging (MRI) scans (7.0T Biospec 70/30, Bruker, BioSpin, Ettlingen, Germany) of the fixed brains (Fig. 2A) and then compared with the rat brain atlas^53^. The brains were first suspended in Fomblin (Sigma-Aldrich, St. Louis, MO, USA) to maximize contrast, and T2-weighted images were acquired in the three anatomical planes with 0.078 × 0.078 mm of voxel size and 0.250 mm of slice thickness.

### Irreversibility computation

We used here LFP recordings, previously resampled at 200 Hz and detrended. The irreversibility was computed under each state in 10-s windows extracted from the time series recorded from all the cortical areas (Fig. 1E, F). In particular, considering two time series *x*(*t*) and *y*(*t*), we computed their reversed backward versions *x*^(*r*)^(*t*) and *y*^(*r*)^(*t*), flipping the time ordering. The causal dependency between two time series was measured by their correlation shifted in time by a time step Δ*t* = *T*:

$$\:C_{{forward}} \left( T \right) = \left\langle {x\left( t \right),y\left( {t + T} \right)} \right\rangle$$

$$\:C_{{backward}} \left( T \right) = \left\langle {x^{{\left( r \right)}} \left( t \right),y^{{\left( r \right)}} \left( {t + T} \right)} \right\rangle$$

We performed a manual selection of the Δ*t* used in this study, whereby we varied the time step from 0.01 s to 0.75 s and observed the decay in the autocorrelation. Our results were consistent against Δ*t* variation in the interval [0.25, 0.75] s. For this reason and to reduce the time required for the computation we choose to use here Δ*t* = 0.75 s.

The pairwise irreversibility value was given by the absolute difference between the causal relationship of the two-time series, in the forward and reversed time evolution:

$$\:{I}_{x,y}\left(T\right)=\left|{C}_{forward}\left(T\right)-{C}_{backward}\left(T\right)\right|$$

Such formalism described in Deco et al.^10^ was adapted here to multidimensional time series to be applied to multielectrode cortical recordings, where the irreversibility is given by the quadratic distance between the forward and reversal time-shifted correlation matrices. We computed both pairwise irreversibility per brain area, the hierarchy of the macroscopic cortical dynamics estimated by the variability of the level of irreversibility across different cortical areas, and the average irreversibility value per state.

### Statistical analysis

In order to test the differences in the irreversibility distributions of the different brain states, and given that these distributions were not normally distributed, the non-parametric statistical test Wilcoxon Rank Sum was chosen, as illustrated in Figs. 3, 4 and 5; Tables 1 and 2. A *p*-value of less than 0.01 was used to define significance.

## Electronic supplementary material

Below is the link to the electronic supplementary material.

Supplementary Material 1

## Data Availability

The datasets generated during and/or analysed during the current study are available from the corresponding author on reasonable request.
