# Supplementary material for: The temporal asymmetry of cortical dynamics as a signature of brain states
Source: Sci Rep. 2024 Oct 16;14:24271. doi: 10.1038/s41598-024-74649-1 (PMC11484927; doi:10.1038/s41598-024-74649-1)
Supplement: Supplementary file 1 — Supplementary Material 1 [file 41598_2024_74649_MOESM1_ESM.docx]

**Supplementary Information (Figures and Tables)**


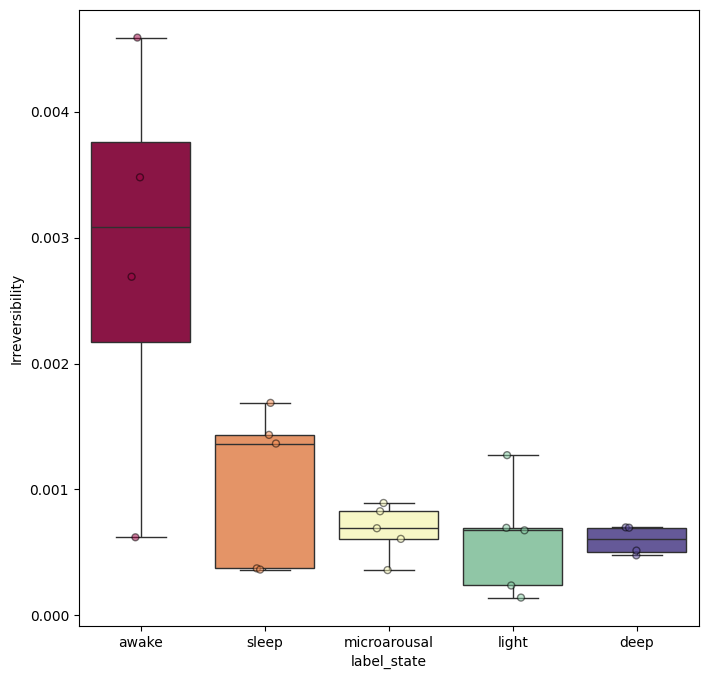


Supplementary Figure 1: Average irreversibility across windows in each state of each animal

| **State 1** | **State 2** | **p-value** | **Significance** |
| --- | --- | --- | --- |
| **awake** | sleep | 5.51041297542823E-17 | significant |
| **awake** | microarousal | 9.33574176323E-28 | significant |
| **awake** | light | 5.95385474208188E-34 | significant |
| **awake** | deep | 1.55317298331253E-45 | significant |
| **sleep** | microarousal | 0.07225991626537400 | not significant |
| **sleep** | light | 0.9938368455990810 | not significant |
| **sleep** | deep | 0.002039332550313710 | significant |
| **microarousal** | light | 0.001947149009189350 | significant |
| **microarousal** | deep | 2.01992952735894E-19 | significant |
| **light** | deep | 1.56483530246387E-08 | significant |

Supplementary Table 1: Wilcoxon Rank Sum with Bonferroni correction on all window values of irreversibility

| **State 1** | **State 2** | **p-value** | **Significance** |
| --- | --- | --- | --- |
| **awake** | sleep | 4.80859886138666E-14 | significant |
| **awake** | microarousal | 1.93986600932699E-22 | significant |
| **awake** | light | 6.24691195664836E-32 | significant |
| **awake** | deep | 6.99384349988566E-41 | significant |
| **sleep** | microarousal | 0.2805847591193940 | not significant |
| **sleep** | light | 0.19612998558132300 | not significant |
| **sleep** | deep | 0.0006493696242357570 | significant |
| **microarousal** | light | 2.61559098221038E-05 | significant |
| **microarousal** | deep | 8.05378826092929E-16 | significant |
| **light** | deep | 0.0001225872533135050 | significant |

Supplementary Table 2: Wilcoxon Rank Sum with Bonferroni correction on all window values of hierarchy

*
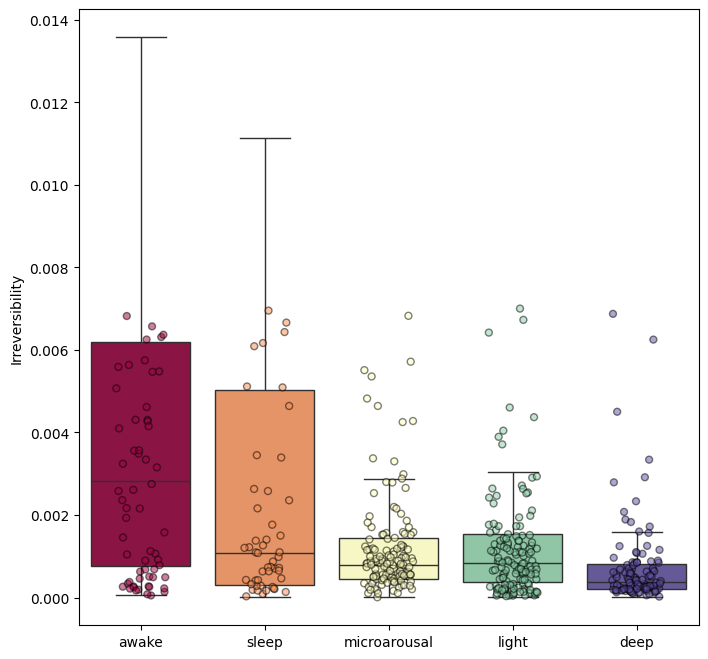
*

Supplementary Figure 2: Irreversibility for each brain state, each dot represents the value for a 20s window

| **State 1** | **State 2** | **p-value** | **Significance** |
| --- | --- | --- | --- |
| **awake** | sleep | 0.001151881863789780 | significant |
| **awake** | microarousal | 2.07510253848239E-17 | significant |
| **awake** | light | 3.34105656508186E-17 | significant |
| **awake** | deep | 3.19507798865644E-27 | significant |
| **sleep** | microarousal | 0.050420427563466600 | not significant |
| **sleep** | light | 0.045838775256456300 | not significant |
| **sleep** | deep | 2.27258511855807E-07 | significant |
| **microarousal** | light | 0.7047794866890970 | not significant |
| **microarousal** | deep | 2.36869699326176E-12 | significant |
| **light** | deep | 7.35139096907107E-11 | significant |

Supplementary Table 3: Wilcoxon Rank Sum with Bonferroni correction for irreversibility values computed on 20s windows


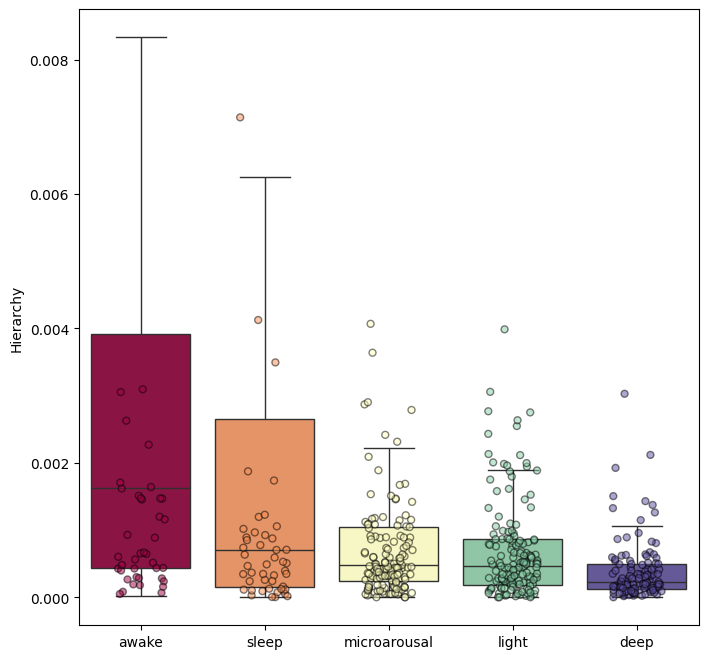


Supplementary Figure 3: Hierarchy for each brain state, each dot represents the value for a 20s window

| **State 1** | **State 2** | **p-value** | **Significance** |
| --- | --- | --- | --- |
| **awake** | sleep | 0.0024691327752300600 | significant |
| **awake** | microarousal | 7.9106172170124E-14 | significant |
| **awake** | light | 3.92594117491836E-16 | significant |
| **awake** | deep | 2.60234144554621E-25 | significant |
| **sleep** | microarousal | 0.01778685251412680 | not significant |
| **sleep** | light | 0.0022228678998051700 | significant |
| **sleep** | deep | 4.56250732511035E-08 | significant |
| **microarousal** | light | 0.17781140146851200 | not significant |
| **microarousal** | deep | 2.20365781165109E-11 | significant |
| **light** | deep | 7.37287885569865E-08 | significant |

Supplementary Table 4: Wilcoxon Rank Sum with Bonferroni correction for hierarchy values computed on 20s windows

*
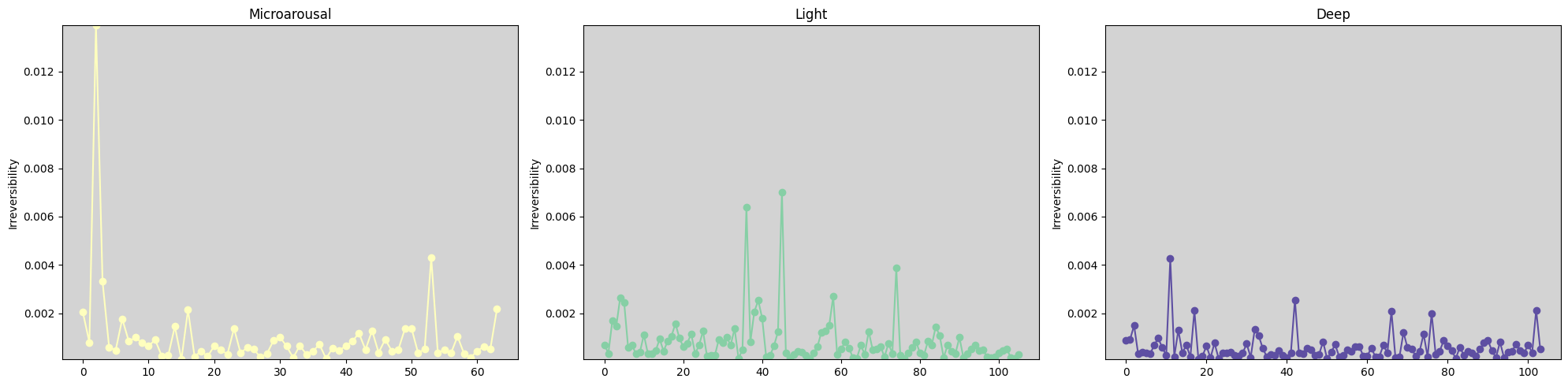
*

Supplementary Figure 4: Temporal changes of Irreversibility in progressive windows of MA, LA and DA from one subject


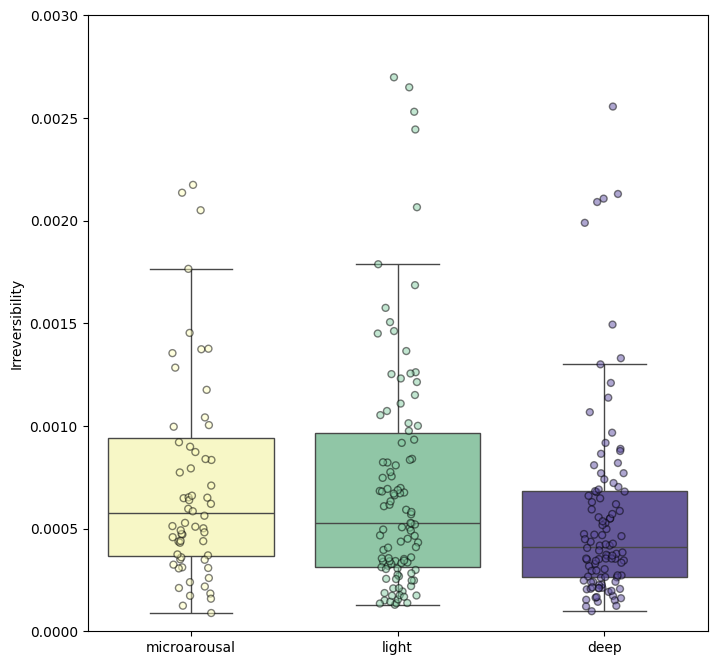


Supplementary Figure 5: Boxplot of Irreversibility in progressive windows of MA, LA and DA from one subject

| **State 1** | **State 2** | **p-value** | **Significance** |
| --- | --- | --- | --- |
| microarousal | light | 0.4772137568135810 | not significant |
| microarousal | deep | 0.005839519877209470 | significant |
| light | deep | 0.03584275716095680 | not significant |

Supplementary Table 5: Wilcoxon Rank Sum with Bonferroni correction for Irreversibility values of consecutive MA, LA and DA windows in one subject

| **Area** | **State** | **Mean** | **STD** |
| --- | --- | --- | --- |
| **prelimbic** | awake | 0.013 | 0.029 |
| **prelimbic** | microarousal | 0.002 | 0.006 |
| **prelimbic** | light | 0.002 | 0.005 |
| **prelimbic** | deep | 0.001 | 0.003 |
| **prelimbic** | sleep | 0.005 | 0.017 |
| **sensory** | awake | 0.024 | 0.04 |
| **sensory** | microarousal | 0.004 | 0.01 |
| **sensory** | light | 0.003 | 0.006 |
| **sensory** | deep | 0.002 | 0.004 |
| **sensory** | sleep | 0.007 | 0.014 |
| **parietal** | awake | 0.012 | 0.023 |
| **parietal** | microarousal | 0.003 | 0.008 |
| **parietal** | light | 0.003 | 0.006 |
| **parietal** | deep | 0.003 | 0.012 |
| **parietal** | sleep | 0.004 | 0.007 |
| **visual** | awake | 0.008 | 0.016 |
| **visual** | microarousal | 0.003 | 0.006 |
| **visual** | light | 0.002 | 0.005 |
| **visual** | deep | 0.003 | 0.012 |
| **visual** | sleep | 0.005 | 0.01 |
| **motor** | awake | 0.006 | 0.02 |
| **motor** | microarousal | 0.001 | 0.002 |
| **motor** | light | 0.001 | 0.001 |
| **motor** | deep | 0.003 | 0.016 |
| **motor** | sleep | 0.002 | 0.012 |

Supplementary Table 6: Average and standard deviation values of Irreversibility for each condition and area.

*
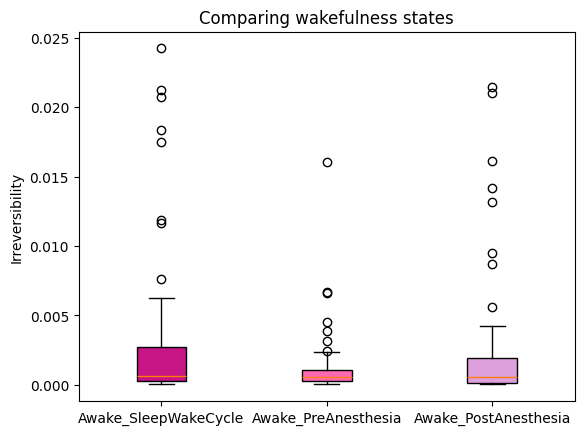
*

Supplementary Figure 6: Irreversibility of Wakefulness periods from the natural-sleep wake cycle, prior to anesthesia induction and after fading out of anesthesia

| **State 1** | **State 2** | **p-value** | **Significance** |
| --- | --- | --- | --- |
| sleep | pre | 0.1584 | not significant |
| sleep | post | 0.5091 | not significant |
| pre | post | 0.8590 | not significant |

Supplementary Table 7: Wilcoxon Rank Sum with Bonferroni correction on all window values of irreversibility


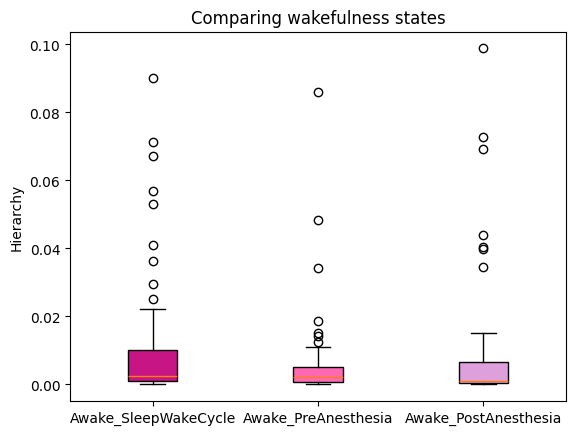


Supplementary Figure 7: Hierarchy of Wakefulness periods from the natural-sleep wake cycle, prior to anesthesia induction and after fading out of anesthesia

| **State 1** | **State 2** | **p-value** | **Significance** |
| --- | --- | --- | --- |
| sleep | pre | 0.0394 | not significant |
| sleep | post | 0.0670 | not significant |
| pre | post | 0.4415 | not significant |

Supplementary Table 8: Wilcoxon Rank Sum with Bonferroni correction on all window values of hierarchy


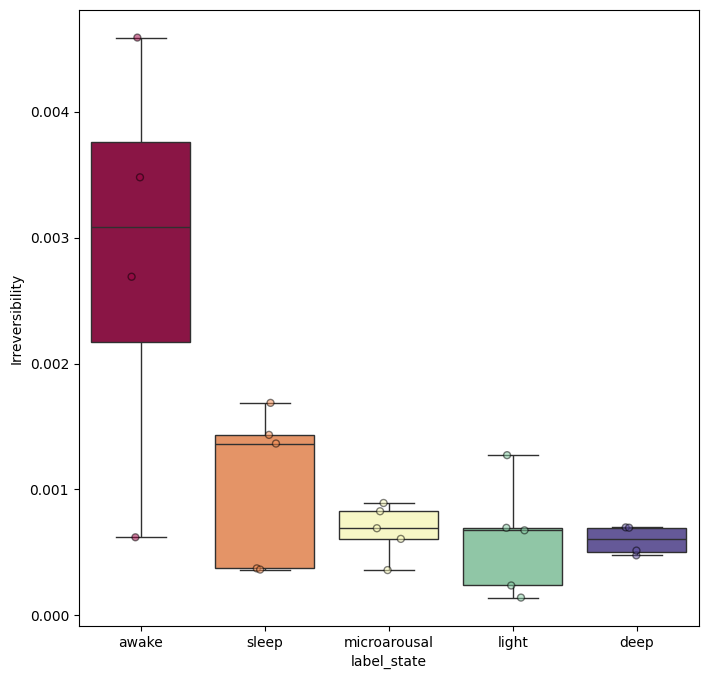


Supplementary Figure 8: Subject averaged group level Irreversibility


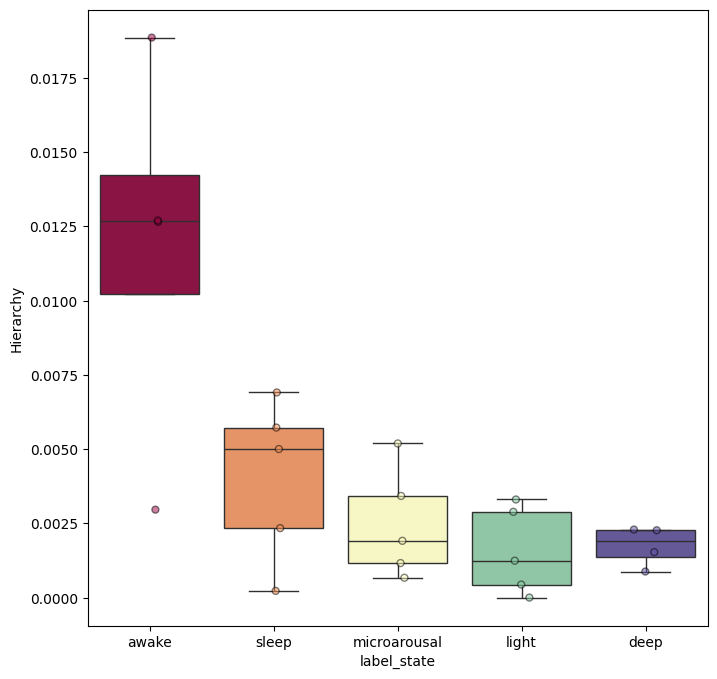


Supplementary Figure 9: Subject averaged group level Hierarchy


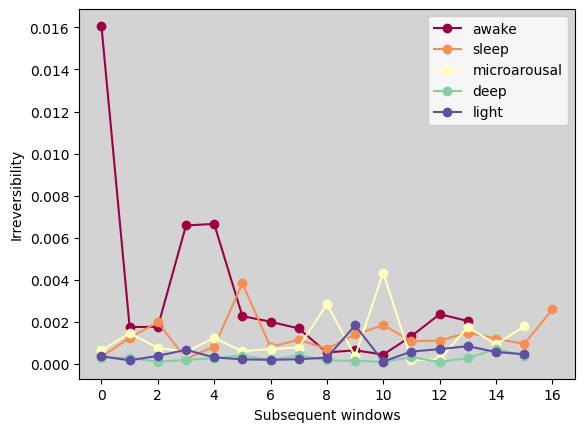


Supplementary Figure 10: Dynamic change of Irreversibility in subsequent windows in one example subject under different states


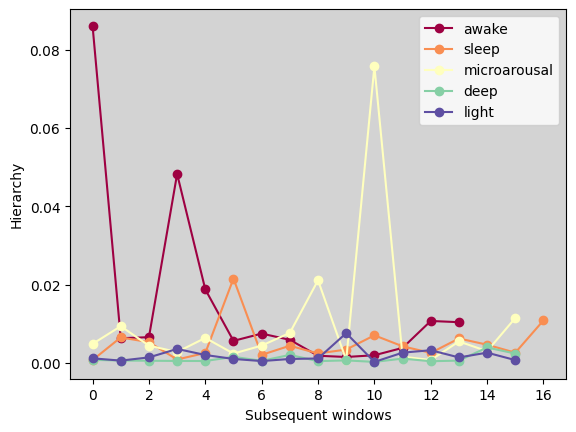


Supplementary Figure 11: Dynamic change of Hierarchy in subsequent windows in one example subject under different states
